# Early Postoperative Atelectasis After Liver Cancer Surgery Affects Exercise Tolerance One Month Post-surgery: A Retrospective Cohort Study

**DOI:** 10.7759/cureus.95121

**Published:** 2025-10-22

**Authors:** Shota Okuno, Kenta Kawamitsu, Kengo Shirado, Yutaro Ohnishi, Taichi Ogami, Takashi Kido, Toshihiro Yamashita, Yo-ichi Yamashita

**Affiliations:** 1 Rehabilitation, Aso Iizuka Hospital Co. Ltd, Iizuka, JPN; 2 Surgery, Aso Iizuka Hospital Co. Ltd, Iizuka, JPN

**Keywords:** 6 minute walk distance, early postoperative atelectasis, hepatocellular carcinoma, liver cancer, postoperative pulmonary complications, rehabilitation

## Abstract

Introduction

Surgical resection is a curative treatment for liver cancer. Postoperative atelectasis is a common complication, but its impact on functional recovery, specifically exercise tolerance, remains unclear. Exercise tolerance, measured by the 6-minute walk test (6MWT), is a key predictor of postoperative outcomes. This study aimed to determine the impact of postoperative atelectasis on the change in exercise tolerance one month after liver cancer surgery.

Methods

This retrospective cohort study included 55 patients undergoing primary liver cancer surgery between 2019 and 2023. The primary outcome was the change in 6-minute walk distance (Δ6MWD) from baseline to one month post-surgery. Atelectasis was diagnosed via CT scan one week postoperatively. We used inverse probability of treatment weighting (IPW) to adjust for confounding variables between the atelectasis and non-atelectasis groups.

Results

Atelectasis was present in 22 of 55 patients (40.0%). The mean Δ6MWD was significantly worse in the atelectasis group (−41.5 m) versus the non-atelectasis group (−8.0 m, p=0.002). After IPW adjustment, which successfully balanced covariates (all standardized mean differences < 0.1), atelectasis remained significantly associated with a greater decline in 6MWD (adjusted coefficient: −32.5, 95% CI: −61.1 to −3.4; p=0.026), exceeding the minimal clinically important difference.

Conclusion

This is the first study to show that postoperative atelectasis significantly impairs exercise tolerance one month after liver cancer surgery. This study is limited by its small sample size, single-center design, and short follow-up period. However, our findings highlight the need for effective atelectasis prevention strategies and suggest that monitoring exercise tolerance is crucial for optimizing postoperative recovery in this population.

## Introduction

Liver cancer is one of the most common cancers worldwide, with more than 900,000 new cases diagnosed annually in 2020 [1]. The curative treatment for liver cancer is surgical resection, and with advances in surgical techniques, the number of patients dying from liver resection has declined [2]. Recent reports indicate that patients with liver cancer are being diagnosed at an older age compared with previous decades [3] and that older adult patients with a history of cancer have lower grip strength, walking speed [4], and exercise tolerance [5] than the general older adult population. Older adult patients are prone to exercise intolerance after surgery [6]. Postoperative exercise tolerance is also a concern after liver cancer surgery. However, most studies on postoperative liver cancer surgery have employed complications and the length of hospital stay as outcomes [7-10]. Notably, a recent review identified the lack of information on physical function before and after surgery for resectable liver cancer [5]. In particular, exercise tolerance, as measured by the 6-minute walk test (6MWT), is reported to be a highly valid assessment of postoperative surgical recovery and construct validity [11,12] and is associated with postoperative quality of life [13,14]. Postoperative exercise tolerance, as measured by the 6MWT, has been reported to recover in the natural course of the disease [14,15], while subsequent recovery of the 6MWT has been reported to be prolonged if the 6MWT has not improved within the first postoperative month [16]. In light of the above, it is important to investigate changes in exercise tolerance over time and modifiable factors in the first month after liver cancer surgery.

On the other hand, postoperative atelectasis is a relatively common pulmonary complication after abdominal and hepatic surgery, frequently reported following liver resection [17,18]. Patients undergoing liver resection are particularly susceptible to early postoperative atelectasis because of their anatomical proximity to the diaphragm and factors such as pleural effusion, secretion retention, and diaphragmatic dysfunction. During hepatectomy, subphrenic manipulation and postoperative drains can elevate the right hemidiaphragm, while reactive pleural effusions secondary to bile leakage or hemorrhage may further compromise pulmonary expansion. Indeed, it has been reported that atelectasis occurs in 30-40% of patients in the early postoperative period after liver cancer surgery [17,18]. Atelectasis is associated with decreased pulmonary function [19] and hypoxemia [20,21]. Because lung hypofunction and hypoxemia are known to reduce exercise tolerance [22,23], the occurrence of atelectasis after liver cancer surgery may hinder postoperative recovery of exercise capacity. While postoperative atelectasis is often considered a transient condition, previous studies suggest that its effects on respiratory function may persist for several weeks [24]. These findings suggest that postoperative respiratory dysfunction, including atelectasis, may not fully resolve in the early postoperative period, potentially impacting exercise tolerance at one month postoperatively. Moreover, impaired ventilatory responses and prolonged oxygen desaturation may limit a patient's ability to engage in early rehabilitation and mobilization, both of which are essential for recovering exercise tolerance. If atelectasis persists beyond the early postoperative phase, it could lead to chronic pulmonary dysfunction, reduced physical activity, and subsequent deconditioning, all contributing to lower exercise tolerance at one month after surgery. Given these factors, postoperative atelectasis may not simply be a transient pulmonary event but could have a prolonged impact on recovery. However, no study has investigated the relationship between postoperative atelectasis and exercise tolerance after liver cancer surgery.

The aim of this study was to investigate postoperative changes in exercise tolerance after liver cancer surgery and to clarify the influence of early postoperative atelectasis on these changes. The specific objectives were: (1) to describe changes in exercise tolerance before and one month after liver cancer surgery, and (2) to examine whether the development of early postoperative atelectasis affects postoperative exercise tolerance.

## Materials and methods

**Figure 1:**
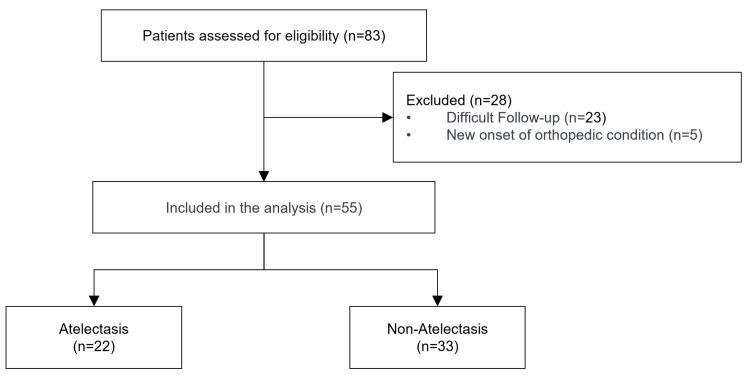
Flow chart of the patient selection process

This retrospective cohort study was conducted at the Iizuka Hospital between November 2019 and November 2023. In this study, at our hospital, patients were selected consecutively if they were diagnosed with and underwent surgery for primary liver cancer during the study period. The exclusion criteria were death within one month after surgery, difficult follow-up, or new orthopedic disease. Difficult follow-up referred to cases in which patients did not undergo the six-minute walk test either at discharge or at the one-month postoperative follow-up. For those included in the analysis, there were no missing data in any of the evaluated variables. Finally, this study included 55 patients who met our eligibility criteria and did not meet the exclusion criteria during the study period (Figure 1). The sample size was determined by balancing the frequency of liver cancer surgery in the hospital with statistical power. Physicians, nurses, dietitians, and physical therapists collaborated to provide perioperative care for patients undergoing primary liver cancer surgery during this period using a clinical path.

Measurements

Measurements were extracted retrospectively from medical records. The measurements included preoperative background factors, such as age, sex, body mass index (BMI), Charlson comorbidity index (CCI), liver cancer stage, Brinkmann index, and preoperative smoking status. By the smoking status, the participants were categorized into three groups: never smoked, former smokers, or current smokers. Intraoperative factors included surgical technique (laparoscopic or open), intraoperative blood loss, and duration of anesthesia. Postoperative factors included the presence or absence of atelectasis, Clavien-Dindo classification [25], type of complications, and the length of hospital stay. The presence or absence of atelectasis was determined by a radiologist based on the results of a computed tomography (CT) scan performed one week postoperatively. Physical therapy evaluations included preoperative and one-month postoperative, the 6-minute walk distance (6MWD), skeletal muscle mass index (SMI), grip strength, 4-m gait speed (4MGS), and sarcopenia. The 6MWD was the distance walked in the 6MWT, which was measured according to the guidelines of the American Thoracic Society [26]. The SMI was measured using bioelectrical impedance analysis (Inbody 770; Inbody Co., Seoul, Korea). Sarcopenia was evaluated using the Asian Working Group for Sarcopenia (AWGS) 2019 criteria [27].

Definition of outcome

The primary outcome was Δ6MWD, defined as the difference between the one-month postoperative and preoperative 6MWD (post-pre). Lower values indicate reduced exercise tolerance, whereas higher values indicate improvement.

Physical therapy programs

All participants started physiotherapy the day after surgery. Walking and chest physiotherapy were administered until postoperative day three. From postoperative day four, exercise therapy was initiated under physician supervision in most patients. The program began with low-intensity resistance and aerobic training (20-30% of maximal load) using a recumbent cycle ergometer and lower-limb training machines. The intensity was gradually increased according to patient tolerance, aiming for 60-70% of the target heart rate calculated using the Karvonen formula [28] and a rating of 2-4 on the modified Borg scale. Even in patients with postoperative drains, exercise therapy was not restricted; the drains were secured to clothing, and exercises were performed as usual. Resistance exercises were performed using standard lower-limb training machines, including recumbent ergometers. The physiotherapy program was continued until discharge from the hospital, with no ongoing physiotherapy after discharge.

Statistical analyses

The retrospective cohort study design relies on historical data and is, therefore, subject to incomplete records and possible selection bias. Therefore, a comprehensive record review and validation of the data by multiple physical therapists were conducted to minimize these biases. For statistical analyses, we first compared the atelectasis and non-atelectasis groups. Continuous variables with normal distributions are described as mean ± standard deviation, and those with non-normal distributions are described as median (interquartile range). Data normality was assessed by visually checking the shape of the distribution of each variable using histograms. The Shapiro-Wilk test was then used to statistically test whether the data conformed to a normal distribution. For comparative analysis for continuous variables, the t-test was used for normality and the Wilcoxon rank sum test for non-normality, while the χ2 test was employed for categorical variables. Normality was assessed for differences between groups. Next, to adjust for potential bias and confounding in group comparisons between the atelectasis and non-atelectasis groups, we performed inverse probability weighted estimation (IPW), a method that estimates the probability (propensity score) associated with each participant's atelectasis status and weights each group based on that score [29]. This process homogenized the background factors of the atelectasis and non-atelectasis groups and improved the validity of comparisons. IPW was employed to effectively adjust for potential selection bias and confounding factors in group comparisons based on the presence or absence of atelectasis in a limited sample size (55 patients). The background factors adjusted by propensity scores were age, sex, CCI, cancer stage, smoking status, preoperative 6MWD, surgical technique, and intraoperative blood loss. To confirm the robustness of our model, we conducted a sensitivity analysis by reducing the number of covariates in the propensity score model. Specifically, we tested two alternative models: (1) a reduced model including only key demographic and functional variable (age, sex, CCI, or preoperative 6MWD), and (2) a second reduced model excluding CCI but retaining surgical factors (age, sex, preoperative 6MWD, and surgical technique). The results showed that the estimated effects remained stable across models, indicating that our original model was well-specified and not overly dependent on any confounder. These factors were selected because of their potential impact on atelectasis and exercise tolerance after liver cancer surgery. The validity of the propensity score model results was assessed by examining whether the absolute value of the standardized difference (SMD) was below the recommended value of 0.1 [29]. In this study, a two-step regression analysis was performed to estimate the effect of the presence or absence of atelectasis on Δ6MWD. First, a single regression analysis was performed using the ordinary least squares (OLS) method. Second, weighted regression analysis was used for the data after the application of IPW to evaluate the impact of atelectasis on Δ6MWD. The statistical analyses were conducted in R (version 4.2.2, R Foundation for Statistical Computing, Vienna, Austria) software. The significance level was set at 5%.

Ethical considerations

This study was conducted in accordance with the Declaration of Helsinki and approved by the Ethics Committee of Iizuka Hospital (approval number: 24004). Clinical data were accessed for research purposes on March 15, 2023. During data collection and analysis, the authors had access to potentially identifiable patient information; however, all data were anonymized before statistical analysis to ensure confidentiality.

## Results

Fifty-five patients met the eligibility criteria and did not meet the exclusion criteria. One week postoperatively, atelectasis occurred in 22 patients (40%). Patients’ mean age was 70.6 ± 9.4 years, there were 37 (67.3%) males, and the average Δ6MWD was -16.0 (-39.5, 2.5) m. Physical function before and one month after surgery was as follows: SMI decreased from 6.8 ± 1.1 kg/m2 preoperatively to 6.6 ± 1.1 kg/m2, grip strength decreased from 26.3 kg to 25.1 kg, 4MGS decreased from 1.18 ± 0.24 m/s preoperatively to 1.08 ± 0.24 m/s, sarcopenia increased from 15 (27.3%) patients preoperatively to 23 (41.8%) patients postoperatively (Table 1).

**Table 1:** Characteristics of study participants n(%). Data are means ± SD. Median (IQR), *p<0.05, **p<0.01 Abbreviations: 4MGS, 4-meter gait speed; 6MWD, 6-Minute Walk Distance; Δ6MWD, Difference in between Preoperative 6MWD and 1-Month Postoperative; BMI, Body mass index; CCI, Charlson Comorbidity Index; Pre, Preoperative; SMI, Skeletal Muscle mass index.

Variable		Overall	Atelectasis	Non-Atelectasis	p-value
		n = 55	n = 22 (40)	n = 33 (60)	
Δ6MWD, m		-16.0 (-39.5, 2.5)	-41.5 (-89.5, -17.5)	-8.0 (-25.0, -10.0)	0.002**
Age, years		70.6 ± 9.4	69.9 ± 7.6	71.1 ± 10.5	0.631
Sex	Men	37 (67.3)	16 (72.7)	21 (63.6)	0.565
BMI, kg/m^2^		24.0 ± 3.4	24.4 ± 2.7	23.7 ± 3.8	0.430
CCI	≥3	10 (18.2)	4 (18.2)	6 (18.2)	1.000
Cancer Stage	Stage Ⅰ	17 (30.9)	6 (27.3)	11 (33.3)	0.449
	Stage Ⅱ	29 (52.7)	13 (59.1)	16 (48.5)	
	Stage Ⅲ	8 (14.5)	2 (9.1)	6 (18.2)	
	Stage Ⅳ	1 (1.8)	1 (4.5)	0 (0.0)	
Brinkman Index		392.0 (0.0, 830.0)	355.0 (0.0, 726.3)	392.0 (0.0, 860.0)	0.929
Smoking Status	Never	24 (43.6)	9 (40.9)	15 (45.5)	1.000
	Former	22 (40.0)	9 (40.9)	13 (39.4)	
	Current	9 (16.4)	4 (18.2)	5 (15.2)	
Pre_6MWD, m		480.6 ± 76.0	489.8 ± 76.1	474.5 ± 76.5	0.470
Pre_SMI, kg/m^2^		6.8 ± 1.1	7.0 ±1.1	6.7 ± 1.2	0.348
Pre_Grip, kg		26.3 (20.4, 32.7)	25.4 (22.5, 31.6)	26.5 (19.7, 32.9)	0.925
Pre_4MGS, m/s		1.18 ± 0.24	1.20 ± 0.29	1.18 ± 0.20	0.787
Pre_Sarcopenia	Yes	15 (27.3)	6 (27.3)	9 (27.3)	1.000
1 month 6MWD, m		456.3 ± 75.3	440.5 ± 68.8	466.8 ± 78.6	0.208
1 month SMI, kg/m^2^		6.6 ± 1.1	6.8 ± 1.0	6.5 ± 1.2	0.435
1 month Grip, kg		25.1 (19.8, 31.3)	24.9 (22.0, 31.4)	26.2 (18.9, 30.2)	0.757
1 month 4MGS, m/s		1.08 ± 0.24	1.05 ± 0.21	1.11 ± 0.26	0.397
1 month Sarcopenia	Yes	23 (41.8)	8 (36.4)	15 (45.5)	0.583
Operative Approach	Open	30 (54.5)	16 (72.7)	14 (42.4)	0.032*
Anesthesia Time, min		343.5 ± 98.9	388.6 ± 86.6	313.4 ± 96.3	0.005**
Operation Time, min		259.1 ± 90.0	298.8 ± 84.6	232.6 ± 84.6	0.006**
Blood Loss, mL		150.0 (50.0, 371.5)	282.0 (86.3, 437.3)	103.0 (40.0, 210.0)	0.031*
Hospital Stay, day		9.0 (8.0, 12.0)	11.0 (9.5, 15.5)	9.0 (8.0, 10.0)	0.002**

Comparing the two groups, Δ6MWD was -41.5 (-89.5, -17.5) m and -8.0 (-25.0, 10.0) m in the atelectasis and the non-atelectasis groups, respectively, and these values were significantly different (p = 0.002). Laparotomy was performed in 16 (72.7%) and 14 (42.4%) patients in the atelectasis and non-atelectasis groups, respectively, which was also significantly different (p = 0.032). The hospital stay was 11.0 (9.5, 15.5) days and 9.0 (8.0, 10.0) days in the atelectasis and non-atelectasis groups, respectively, and was significantly longer in the atelectasis group (p = 0.002) (Table 1). The percentage of patients with Clavien-Dindo classification grade II or more was seven (31.8%) in the atelectasis group and four (12.1%) in the non-atelectasis group, but this difference was not statistically significant. No other factors differed significantly between the two groups (Table 2).

**Table 2:** Postoperative complications n (%)

Variable		Overall	Atelectasis	Non-Atelectasis	p-value
		n = 55	n = 22 (40)	n = 33 (60)	
Clavien-Dindo Classification	Grade Ⅰ	6 (10.9)	4 (18.2)	2 (6.1)	0.081
	Grade Ⅱ	8 (14.5)	5 (22.7)	3 (9.1)	
	Grade Ⅲ	2 (3.6)	1 (4.5)	1 (3.0)	
	Grade Ⅳ	1 (1.8)	1 (4.5)	0 (0.0)	
Clavien-Dindo Classification	≧Ⅱ	13 (23.6)	7 (31.8)	4 (12.1)	0.094
Anastomotic Leakage	yes	0 (0)	0 (0)	0 (0)	NA
Liver Failure	yes	0 (0)	0 (0)	0 (0)	NA
Liver Abscess	yes	1 (1.8)	1 (4.5)	0 (0.0)	0.400
Delirium Diagnosed	yes	0 (0)	0 (0)	1 (3.0)	1.000
Pneumonia	yes	1 (1.8)	1 (4.5)	0 (0.0)	0.400
Portal Vein Thrombosis	yes	5 (9.1)	2 (9.1)	3 (9.1)	1.000
Lower Limb Thrombosis	yes	1 (1.8)	0 (0)	1 (3.0)	1.000
Internal Carotid Artery Thrombosis	yes	3 (5.4)	3 (13.6)	0 (0.0)	0.059
Ascites	yes	2 (3.6)	2 (9.1)	0 (0.0)	0.400
Pseudomembranous Colitis	yes	0 (0)	0 (0)	0 (0)	NA
Ileus	yes	2 (3.6)	2 (9.1)	0 (0.0)	0.156
Transverse Colon Perforation	yes	1 (1.8)	0 (0)	1 (3.0)	1.000
Hemorrhage	yes	0 (0)	0 (0)	0 (0)	NA
Biliary Fistula	yes	1 (1.8)	1 (4.5)	0 (0.0)	0.400

The absolute values of the SMD of the background factors were all less than 0.1 after IPW, confirming that the groups were well balanced after IPW (Table 3). To evaluate the impact of covariate selection, we conducted a sensitivity analysis using different models after applying IPW (Table 4). In the IPW-Base Model, the regression coefficient was β = -32.5 (95% CI: -61.1, -3.4, p = 0.026). In contrast, the IPW-Model1 showed β = -38.0 (95% CI: -67.0, -9.3, p = 0.010), and the IPW-Model2 resulted in β = -31.0 (95% CI: -58.0, -3.7, p = 0.027). The β coefficients and confidence intervals were similar across the three models, indicating that modifying the covariate selection did not lead to substantial changes in the estimated treatment effect. In a single regression analysis before IPW, atelectasis had a significant negative effect on Δ6MWD (regression coefficient -41.6, 95% confidence interval -67.7, -15.5, p = 0.002). In a single regression analysis after the application of IPW with balanced background factors, the effect atelectasis had a significant negative impact on Δ6MWD (regression coefficient -32.5, 95% confidence interval -61.1, -3.4, p=0.026) (Table 5).

**Table 3:** Background factors before and after Inverse Probability Weighting (IPW) n (%). Data are means ± SD. Median (IQR) Abbreviations: 6MWD, 6-Minute Walk Distance; ESS: Effective Sample Size, calculated to reflect the true statistical power of the weighted sample; CCI, Charlson Comorbidity Index; SMD, Standardized Mean Difference.

		Before IPW							After IPW					
		Atelectasis		Non-Atelectasis		SMD			Atelectasis		Non-Atelectasis		SMD	
		n = 22		n = 33					ESS = 54		ESS = 54			
Age, year		69.9 ± 7.6		71.1 ± 10.5		0.13			70.1 ± 7.7		70.7 ± 11.0		0.07	
Sex	Men	16 (72.7)		21 (63.6)		0.09			33.3 (61.5%)		33.0 (60.6%)		0	
CCI	≥3	4 (18.2)		6 (18.2)		0			8.2 (15.2%)		12.3 (22.6%)		0.07	
Cancer Stage	Stage Ⅰ	6 (27.3)		11 (33.3)		0.06			16.8 (31.0%)		16.9 (31.0%)		0	
	Stage Ⅱ	13 (59.1)		16 (48.5)		0.1			30.0 (55.5%)		29.7 (54.6%)		0.01	
	Stage Ⅲ	2 (9.1)		6 (18.2)		0.09			6.3 (11.6%)		7.8 (14.4%)		0.03	
	Stage Ⅳ	1 (4.5)		0 (0.0)		0.05			1.0 (1.8%)		0.0 (0.0%)		0.02	
Smoking Status	Never	9 (40.9)		15 (45.5)		0.05			23.5 (43.4%)		25.5 (46.9%)		0.03	
	Former	9 (40.9)		13 (39.4)		0.02			20.0 (36.9%)		19.6 (36.0%)		0.01	
	Current	4 (18.2)		5 (15.2)		0.03			10.6 (19.6%)		9.3 (17.1%)		0.03	
Preoperative 6MWD, m		489.8 ± 76.1		474.5 ± 76.5		0.2			488.3 ± 77.3		481.1 ± 72.8		0.09	
Operative Approach	Open	16 (72.7)		14 (42.4)		0.3			29.5 (54.5%)		29.2 (53.6%)		0	
Blood Loss, mL		282.0 (86.3, 437.3)		103.0 (40.0, 210.0)		0.41			163.5 (50.0, 401.0)		113.8 (41.6, 208.0)		0.07	

**Table 4:** Sensitivity analysis of inverse probability weighting models *p<0.05 Abbreviations: β, Regression Coefficient; CI, Confidence Interval; IPW, Inverse Probability Weighting.

		β	95% CI		p-value
			Lower	Upper	
Base Model	After IPW	-32.5	-61.1	-3.4	0.026*
Model1	After IPW	-38.0	-67.0	-9.3	0.010*
Model2	After IPW	-31.0	-58.0	-3.7	0.027*

**Table 5:** Relationship Between Postoperative One-Week Atelectasis and Δ6MWD *p<0.05, **p<0.01 Abbreviations: Δ6MWD, Difference in 6-minute walking distance before and one month after surgery; β, Regression Coefficient; CI, Confidence Interval; IPW, Inverse Probability Weighting.

	β	95% CI		p-value
		Lower	Upper	
Before IPW	-41.6	-67.7	-15.5	0.002**
After IPW	-32.5	-61.1	-3.4	0.026*

## Discussion

This study revealed two main findings. First, the 6MWD at one month after liver cancer surgery was 16.0 m lower than preoperatively. Second, postoperative atelectasis affected Δ6MWD at one month after surgery.

The 6MWD at one month postoperatively for liver cancer was -16.0 m lower than its preoperative value. Pecorelli et al. [12] reported that 6MWD decreased by approximately 20 m at one month after colorectal cancer surgery, which is comparable to our findings in patients with liver cancer. This similarity suggests that postoperative functional decline in exercise tolerance may share common mechanisms between colorectal and liver cancer surgery. However, patients with liver cancer are reportedly prone to metabolic abnormalities, including increased gluconeogenesis and protein catabolism [30-32]. In the present study, a slight decrease in SMI and grip strength was observed compared to preoperative values. Given that muscle wasting and strength loss are progressive processes, it is important to conduct long-term follow-up studies to assess whether reductions in exercise tolerance one month postoperatively contribute to subsequent declines in muscle mass and strength in patients with liver cancer.

Second, the postoperative atelectasis after liver cancer surgery affected exercise tolerance at one month postoperatively. Ko et al. [33] reported that atelectasis after standby non-cardiothoracic surgery was associated with the length of hospital stay. In the present study, along with the association with the length of hospital stay, a new association with exercise tolerance at one month was demonstrated. Furthermore, the association with exercise tolerance did not change after adjusting for the balance of background factors using IPW. The observed difference in 6MWD (-41.5 m vs. -8.0 m) exceeds the minimum clinically important difference (MCID) range (14.0-30.5 m) in the systematic review by Bohannon and Crouch [34]. This suggests that postoperative atelectasis may have a clinically significant impact on functional recovery. However, as the MCID specific to patients undergoing liver cancer surgery has not been established, further studies are needed to confirm the clinical relevance of our findings in this specific population.

A possible mechanism by which postoperative atelectasis leads to reduced exercise tolerance is that the development of atelectasis often necessitates additional chest physiotherapy to address respiratory complications in the early postoperative period. As a result, the time and intensity available for exercise therapy may become limited, making it difficult to maintain an adequate training level. Atelectasis may also impair exercise tolerance through physiological mechanisms: the collapse of alveoli decreases lung compliance and ventilation efficiency, leading to reduced oxygen transport capacity and increased respiratory workload during exercise. These therapeutic and physiological factors together contribute to early fatigue and diminished exercise performance after surgery. Factors reported to influence postoperative exercise tolerance include early postoperative ambulation [35], early resistance training [36], and continued pulmonary rehabilitation after discharge [37]. We believe that after liver cancer surgery, it is essential to establish a perioperative care program that balances chest physiotherapy and exercise therapy to prevent atelectasis and promote early recovery of exercise tolerance, including after discharge.

This study has several limitations. First, the sample size was relatively small, and the retrospective single-center design may limit the generalizability of the findings. Further prospective studies with increased sample sizes are expected to validate these results. Second, baseline pulmonary function tests were not performed; however, preoperative six-minute walk distance was included as a covariate to partially adjust for baseline differences in exercise and pulmonary capacity. Third, postoperative factors such as biochemical parameters, adherence to physiotherapy, and variability in pulmonary care were not assessed, which may have influenced postoperative recovery. Fourth, in this retrospective analysis, only binary information (presence or absence) was available in the medical records; therefore, severity-based classification was not possible. In addition, the presence or absence of atelectasis was determined by a single reader, which may introduce classification bias. Future prospective studies should incorporate dual-reader or consensus adjudication to enhance reproducibility. Finally, because this study relied on retrospective data, advances in treatment and perioperative management during the study period may not be fully reflected. Despite these limitations, we believe that our study reveals an important association between postoperative atelectasis and exercise tolerance after liver cancer surgery and provides useful insights for future clinical research.

To our knowledge, this is the first study demonstrating that atelectasis after liver cancer surgery affects exercise tolerance. Notably, we were able to demonstrate the association between temporal change and atelectasis after liver cancer surgery by employing the 6MWD, which has high validity as a measure of postoperative recovery. These results emphasize the importance of preventing atelectasis and continuously observing exercise tolerance in the management of patients after liver cancer surgery to help improve future perioperative care programs.

## Conclusions

To our knowledge, this is the first study demonstrating that atelectasis after liver cancer surgery affects exercise tolerance. Notably, we were able to demonstrate the association between temporal change and atelectasis after liver cancer surgery by employing the 6MWD, which has high validity as a measure of postoperative recovery. These results emphasize the importance of preventing atelectasis and continuously observing exercise tolerance in the management of patients after liver cancer surgery to help improve future perioperative care programs.
